# Implementation process and challenges of index testing in Côte d’Ivoire from healthcare workers’ perspectives

**DOI:** 10.1371/journal.pone.0280623

**Published:** 2023-02-08

**Authors:** Nancy Mugisha, Fatoumata Tirera, Naraba Coulibaly-Kouyate, William Aguie, Yao He, Kathryn Kemper, Julia Robinson, Luc N’Goran, Moïse Tuho, Seydou Kouyate, Yacouba Doumbia, Stephen Gloyd, Ahoua Kone

**Affiliations:** 1 Department of Internal Medicine, University of Washington, Washington, Seattle, United States of America; 2 Institut de Recherche et d’Actions en Afrique (IRAA), Abidjan, Côte d’Ivoire; 3 Health Alliance International (HAI), Washington, Seattle, United States of America; 4 Department of Global Health, University of Washington, Washington, Seattle, United States of America; University of Ghana College of Health Sciences, GHANA

## Abstract

A major limiting factor in combatting the HIV epidemic has been the identification of people living with HIV. Index testing programs were developed to face that challenge. Index testing is a focused HIV testing service approach in which family members and partners of people living with HIV are offered testing. Despite the implementation of index testing, there is still a gap between the estimated number of people living with HIV and those who know their status in Côte d’Ivoire. This study aimed to understand the implementation process of index testing in Côte d’Ivoire and to identify implementation challenges from healthcare workers perspectives. In January and February 2020, we conducted a qualitative study through 105 individual semi-structured interviews regarding index testing with clinical providers (physicians, nurses, and midwives) and non-clinical providers (community counselors and their supervisors) at 16 rural health facilities across four regions of Côte d’Ivoire. We asked questions regarding the index testing process, index client intake, contact tracing and testing, the challenges of implementation, and solicited recommendations on improving index testing in Côte d’Ivoire. The interviews revealed that index testing is implemented by non-clinical providers. Passive referral, by which the index client brought their contact to be tested, and providers referral, by which a healthcare worker reached out to the index client’s contact, were the preferred contact tracing and testing strategies. There was not statistically significant difference between immediate and delayed notification. Reported challenges of index testing implementation included index cases refusing to give their partner’s information or a partner refusing to be tested, fear of divorce, societal stigma, long distances, lack of appropriate training in index testing strategies, and lack of a private room for counseling. The recommendations given by providers to combat these was to reinforce HIV education among the population, to train healthcare workers on index testing strategies, and to improve infrastructure, transportation, and communication resources. The study showed that the elements that influenced the process of index testing in Côte d’Ivoire were multifactorial, including individual, interpersonal, health systems, and societal factors. Thus, a multi-faceted approach to overcoming challenges of index testing in Côte d’Ivoire is needed to improve the yield of index testing.

## Introduction

Despite substantial progress in HIV care in the last 30 years, HIV remains a significant problem in most countries of Sub-Saharan Africa (SSA). In 2019, 17.5% of total Disability-Adjusted Life Years (DALYs) within the age group of 15–49 in SSA were attributable to HIV alone [1]. In 2014, as an effort to fast-track the end of the AIDS epidemic, the Joint United Nations Program on HIV and AIDS (UNAIDS) released 2030 targets: 95% of people living with HIV (PLHIV) knowing their status, 95% of people who know their status accessing treatment and 95% of people on treatment achieving viral suppression by 2030 [2].

Achieving the first target has been particularly challenging, many countries -including Côte d’Ivoire -have difficulties identifying PLHIV as reflected by countries reports on the three targets [3]. The UNAIDS 2022 update suggests insufficient political will, frail health systems, weak support for community organizations, and a continued reliance on user fees for health services as some of the problems hindering the achievement of the first 95% target [3]. One of the proposed solutions to achieving the first 95% target is index testing. It is a focused HIV testing service approach in which family members and sexual partners of known PLHIV are offered testing [4]. It is sometimes referred to as assisted partner notification or assisted partner services. PLHIV who have been identified and are in treatment are called index clients or index cases in this context.

Due to the poor identification of cases in Côte d’Ivoire, index testing was introduced in 2015. It was initially implemented as a family-centered approach to HIV care, focusing on the index case’s spouse and children, and was later scaled up to include other sexual partners [5]. The Ministry of Health and Public Hygiene (MSHP) of Côte d’Ivoire partnered with the U.S. President’s Emergency Plan for AIDS Relief (PEPFAR) to scale up index testing through several non-governmental organizations. As of 2020, Health Alliance International (HAI) and the Institute for Research and Action in Africa (IRAA) were among several organizations that worked with PEPFAR and the MSHP to carry out index testing programs.

Index testing has been proven effective in increasing the detection and testing of biological children and sexual partners of PLHIV in various other contexts [6–9]. In Nigeria, Katbi et al. showed the effectiveness of index testing implementation as it contributed to high rates of disclosure (68%), partner tracing (98%), and partner testing (85%) [10]. Numerous implementation outcomes studies have also shown that index testing is feasible, acceptable, and cost-effective in SSA [11–16]. In addition, index testing programs have increased the yield of newly diagnosed HIV cases compared to traditional testing services [13,17,18]. A study of a partner services program in Rwanda demonstrated a positivity yield that was 10 times higher than prior passive testing modalities [18]. A similar study in Uganda had a positivity yield that was 4.5 times the national HIV prevalence [13].

In order to implement index testing services strategically, the MSHP of Côte d’Ivoire recommended four referral strategies: *passive referral* (the index client asked or brought their partner to the health center to be tested); *provider’s referral* (a healthcare worker reached out to the index client’s contact to encourage them to get tested); *referral by contract* (a healthcare worker agreed with the index client on a deadline for them to bring their contact to be tested); and *dual referral* (a healthcare worker and an index client worked together to disclose and ask a contact to be tested) [4,19].

The strategies employed in Côte d’Ivoire for contact tracing and testing are similar to those used in other low and middle-income countries of Africa and Asia. Many studies have shown that using these strategies improves the detection and testing of PLHIV contacts. A behavioral skills training of healthcare workers (HCW) using the passive referral strategy in Malawi showed an increase in the mean number of sexual partners listed per facility per month (pre-training = 6.3, post-training = 10.6) as well as an increase in the number of contacts who received HIV testing (pre-training = 11.1, post-training = 24.8) [20].

A South African study evaluating index testing demonstrated that 51% of PLHIV contacts were tested through referral by contract, 51% by the providers’ referral, and 24% by passive referral [21]. In Central Asia, an assisted partner notification program consisting of contract referral, provider referral, and dual referral increased the number of partners tested per index case recruited (0.5 to 1.4). The number of index cases needed to find one HIV-positive partner decreased significantly (27.4 to 8.3), again proving index testing to be a great tool in achieving the UNAIDS first 95% goal [22].

Even though index testing was introduced in 2015 in Côte d’Ivoire, the rate of identification of PLHIV remained low. The estimated absolute prevalence of HIV in Côte d’Ivoire is 380,000 PLHIV. According to a 2018 population-based HIV impact assessment in Côte d’Ivoire, only 37.2% of surveyed adults who were HIV positive knew their status, or an estimated 140,600 knew their status, by extrapolation from that survey [23].

This study aimed to evaluate HCW’s practices surrounding the index testing process from the time of diagnosis to contact testing, to identify challenges faced at clinical sites that have hindered index testing success, and to gather HCW’s recommendations on how to improve index testing in Côte d’Ivoire. It was conducted at government clinical sites supported by HAI and IRAA.

## Materials and methods

### Study design

This is a qualitative study using semi-structured interviews that examines the implementation process of index testing in Côte d’Ivoire, the challenges of implementation, and the providers’ recommendations on index testing.

### Study setting

The study sites were 16 rural health centers located across four regions of North-central and Eastern Côte d’Ivoire (Gbeke-Hambol, Worodougou-Bere, Bounkani-Gontougou, and Indenie-Djuablin). The sites were chosen because they were priority sites identified by the MSHP and PEPFAR, with large numbers of PLHIV.

### Study population and sample size

We conducted semi-structured interviews with clinical (physicians, nurses, and midwives) and non-clinical providers (community counselors and their supervisors); and completed a total of 105 individual interviews, including 58 with community counselors, 10 with site supervisors, 18 with physicians, 12 with nurses, and seven with midwives. The data was collected in January and February 2020.

### Materials

We used two different interview guides, one template was designed for clinical providers, while the second was for non-clinical providers (S1 File). The interview guides included open and closed-ended questions. The interview template for clinical providers inquired about the degree of involvement of physicians, nurses, and midwives in the index testing program and perceived challenges and facilitators of index testing. The template for non-clinical providers explored five subjects: the intake of a person newly diagnosed with HIV, the process of obtaining names of potential index cases’ contacts (family members and sexual partners), the implementation of referral strategies to test contacts, the challenges of index testing implementation, and the providers recommendations on index testing implementation. We recorded the interviews with a digital voice recorder and field memos.

### Data analysis

We conducted statistical tests for significant difference between pairs of sample means and ANOVA to test for statistically significant differences across multiple sample means.

### Ethical considerations

Through the Global Opportunities Health Fellowship of the University of Washington, we collected the data as a program evaluation for PEPFAR/HAI and the MSHP. We consulted with the University of Washington’s Human Subjects Division, which relayed that ethical approval was not required due to the nature of the study being a program evaluation. We received verbal informed consent from all participants, witnessed by an HAI or IRAA agent. None of the participants refused to be interviewed, but one participant refused to be recorded, they were comfortable with field memos only, which was accommodated during that interview.

## Results

The main findings of the interviews were that two of the four referral strategies are preferred and used (the passive and providers referrals}, that clinical providers have limited involvement in index testing, that there is no significant difference in the practice of immediate or delayed notification, and that the challenges of implementation are about stigma, marital problems, infrastructures, lack of training, long distances and knowledge on HIV. The results on the implementation process were obtained from non-clinical providers only, because clinical providers were not involved in these procedures, but the results on challenges and recommendations for improvement of the program are themes gathered from clinical and non-clinical providers.

### Referral strategies

**Fig 1:**
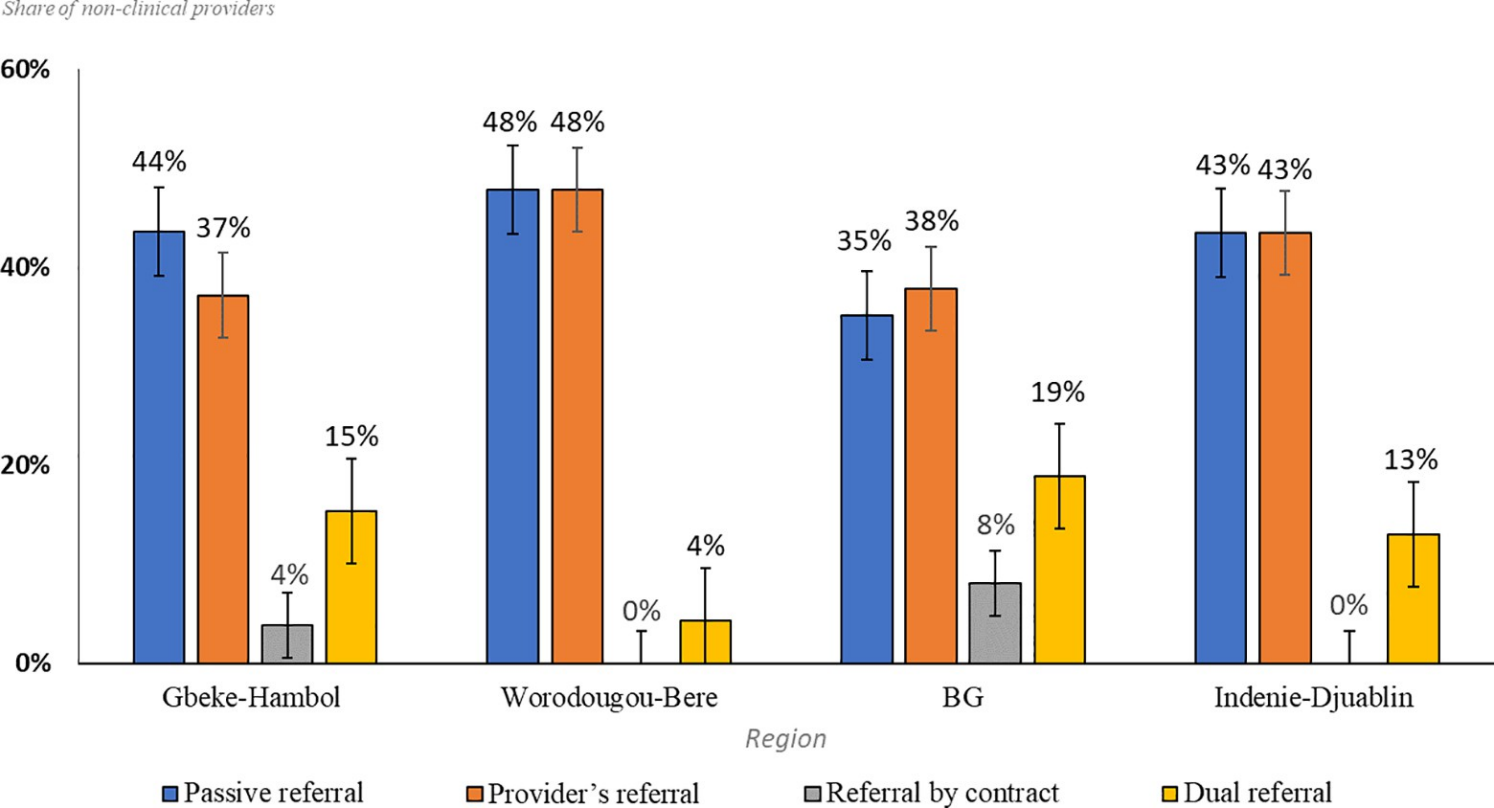
Which strategy do you most often use? Most utilized implementation strategy of contact tracing/testing strategies by region.

We found similar results across all four regions regarding index testing referral strategies used. Interview respondents reported using *passive* and *provider referral* as the main strategies in all regions. 43% of non-clinical providers reported using the *passive referral* strategy, and 41% reported using the *providers’ referral* strategy We also asked if there was one preferred strategy that they used most often, results of which are shown in Table 1 and Fig 1. An ANOVA test comparing the means of preferred referral methods across the four regions in the study is shown in Table 1. The results in Table 1, F = 60.38, p < 0.05 show that passive referral and provider referral have statistically significant higher mean preference rates than the other strategies across all regions. A visual comparison of the means between passive and providers referrals in Fig 1 shows that passive referral has higher preference rates in Gbeke-Hambol while providers referral has a higher preference rates in Bounkani-Gontougou, without statistical significance as the confidence intervals overlap.

**Table 1:** One way ANOVA test for preferred referral strategies.

**A. SUMMARY**						
**Groups**	**Count**	**Sum**	**Average**	**Variance**		
**Passive referral**	4	1.700292	0.425073	0.002825		
**Provider’s referral**	4	1.663217	0.415804	0.002533		
**Referral by contract**	4	0.119543	0.029886	0.001494		
**Dual referral**	4	0.516948	0.129237	0.003852		
**B. ANOVA**						
**Source of Variation**	**SS**	**df**	**MS**	**F**	**P-value**	**F crit**
**Between Groups**	0.484702	3	0.161567	60.37931	0.00	3.490295
**Within Groups**	0.03211	12	0.002676			
**Total**	0.516813	15				

### Clinical providers involvement

**Fig 2:**
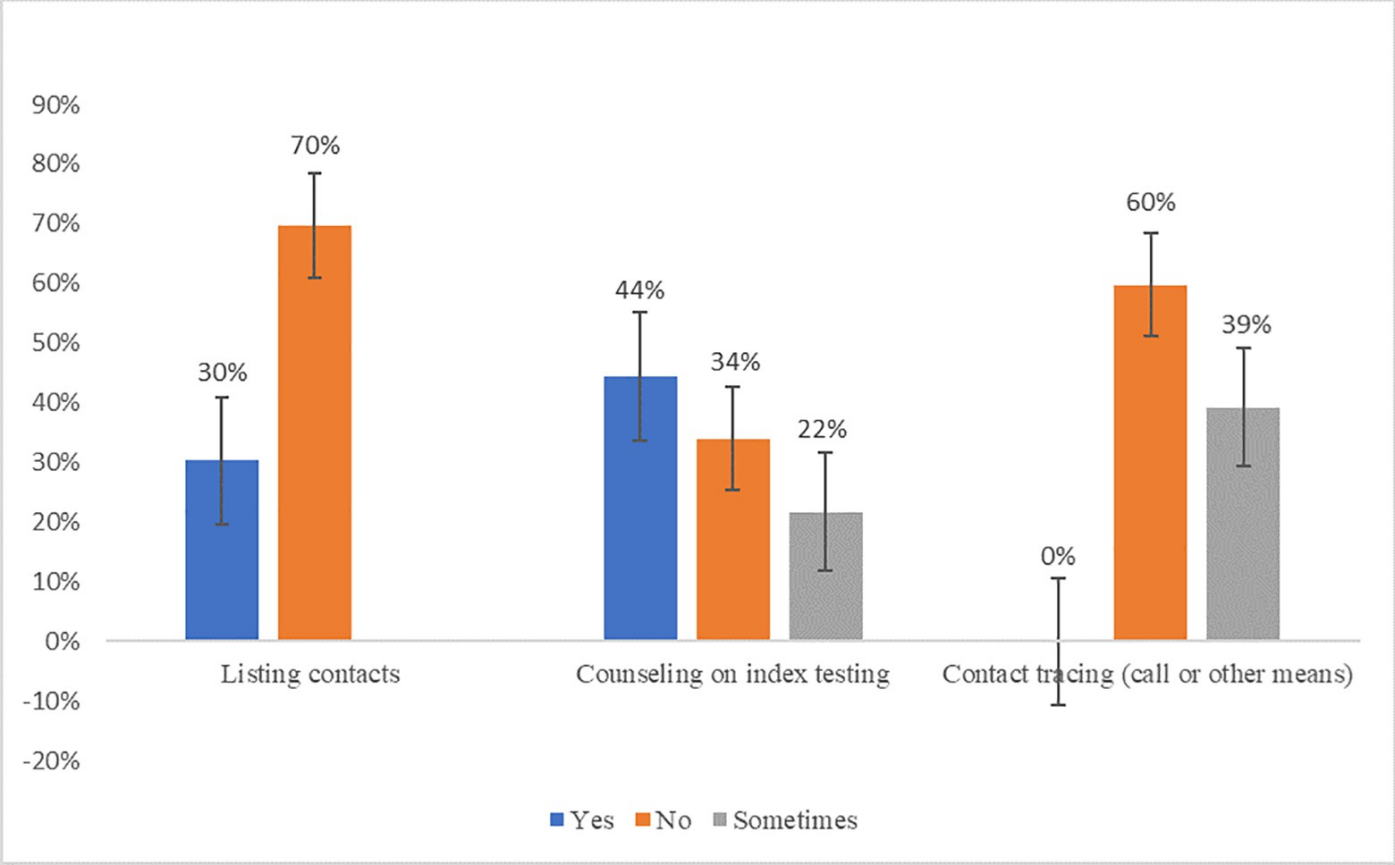
Clinical providers involvement in index testing. Responses on index clients counseling, listing contacts and tracing contacts.

The interviews revealed a limited involvement of clinical providers (physicians, nurses, and midwives) in index testing. Fig 2 shows the responses about index testing involvement. The clinical providers were less likely to be involved in listing and tracing contacts. In both cases, the 95% confidence interval for the likelihood of providers involvement does not overlap with the likelihood of non-involvement. The main contribution that clinical providers reported was counseling index clients during their clinical visits about the importance of testing their contacts.

### Immediate and delayed notification

Regarding initiation of the index testing process, an average of 80% of non-clinical interview participants across the regions reported asking about the patient’s family and sexual partners during the first visit and 88% reporting asking during subsequent visits. However, these rates are not statistically distinguishable from each other at the 95% confidence level (S1 Fig).

Interview participants relayed that asking during the first visit was hard due to the shock of a new diagnosis, and for that reason sometimes they delayed the initiation of contact tracing to subsequent visits. Some providers reported always deferring the registration of family members and sexual partners to subsequent visits.

### Challenges of index testing implementation

**Fig 3:**
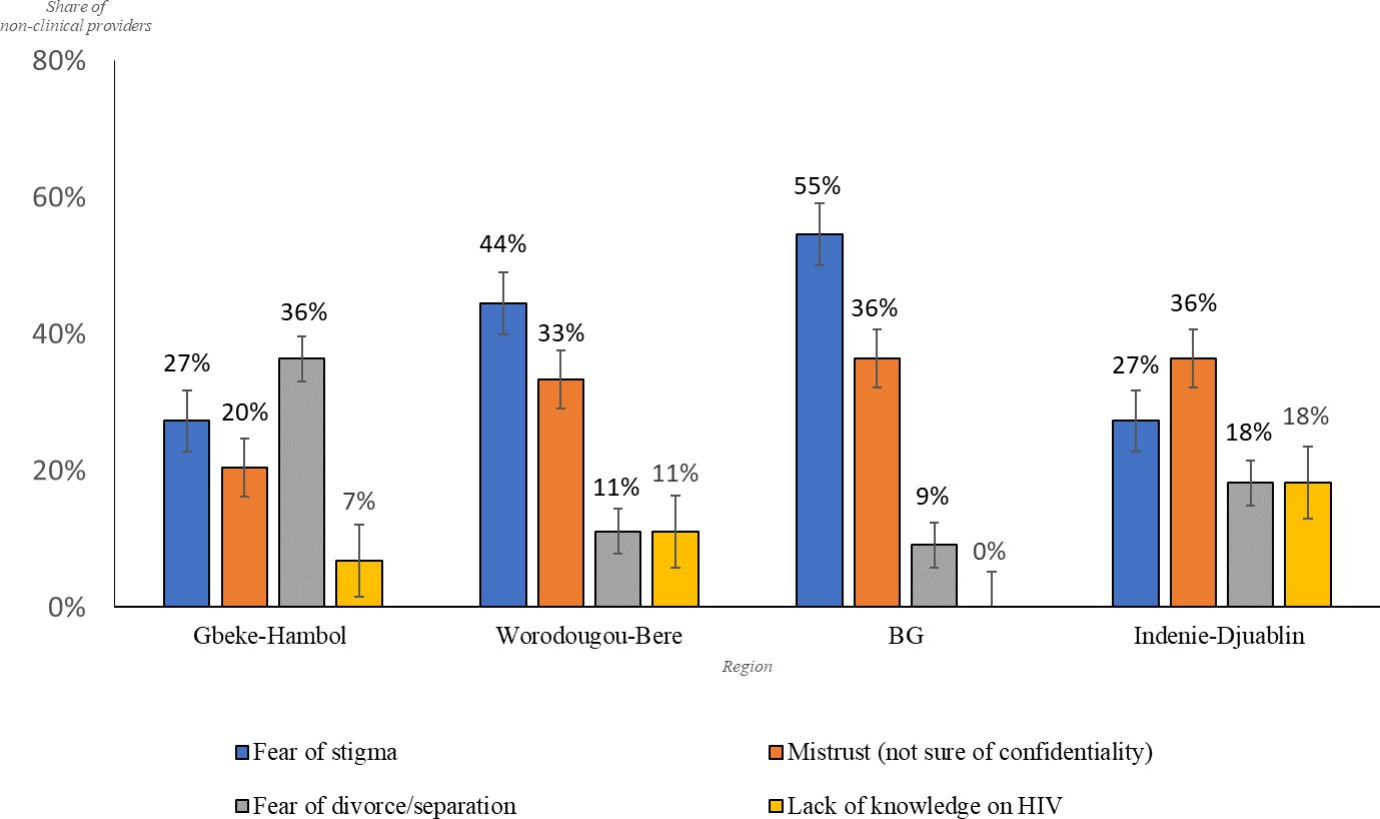
What challenges have you encountered with obtaining the names of family and sexual partners? Challenges faced when obtaining the names of family and sexual partners.

Challenges in identifying contacts reported by the interview respondents reflected patients’ perceptions and attitudes towards HIV: fear of HIV-related stigma and divorce/separation, mistrust of confidentiality in the healthcare system, and lack of knowledge about HIV (Table 2, Fig 3). An ANOVA test comparing the means of the challenges encountered by clinical providers across the four regions in the study is shown in Table 2. The results in Table 2, F = 6.1, p<0.05, show that fear of stigma and mistrust in the healthcare system due to issues of confidentiality are perceived as the most prominent challenges with statistical significance.

**Table 2:** One way ANOVA test for challenges of implementation.

**A. SUMMARY**						
***Groups***	***Count***	***Sum***	***Average***	***Variance***		
**Fear of stigma**	4	1.535354	0.383838	0.018161		
**Mistrust (not sure of confidentiality)**	4	1.265152	0.316288	0.005754		
**Fear of divorce/separation**	4	0.747475	0.186869	0.015407		
**Lack of knowledge on HIV**	4	0.361111	0.090278	0.005817		
**B. ANOVA**						
***Source of Variation***	***SS***	***df***	***MS***	***F***	***P-value***	***F crit***
**Between Groups**	0.206698	3	0.068899	6.105531	0.009162	3.490295
**Within Groups**	0.135417	12	0.011285			
**Total**	0.342114	15				

The quotes below by community counselors reflect a repeated theme of fear of marital consequences or societal stigma:

*"The fear, because sometimes the women are worried that since they were the first one to be tested positive if their husband finds out, he might think that they brought the disease in the home and they [the women] tell themselves: ’if my husband learns about my status, he will divorce me.‴*

*"Sometimes the women are worried about us directly going to their homes because it would be too obvious. In some cases, we organize a community outreach in a neighborhood to talk about HIV and test people while knowing that we are targeting a specific spouse or sexual partner in the neighborhood."*

Interview respondents also noted that many facilities do not have a designated–or confidential -room for counseling. Community counselors in those facilities met with index clients in shared rooms where other community counselors were also working, presenting a tremendous barrier due to a lack of privacy and was a significant part of the mistrust in confidentiality noted above.

After the contacts were identified, the interview participants expressed that some common challenges of testing the identified contacts included: the subjects’ refusal to be tested; patients transferring to another facility or not following up, which made it hard to reach their family members and sexual partners; and long distances that discouraged home visits or discouraged the subjects from coming to the health centers as reflected by the quote below from a community counselor.

*"Our patients live in remote villages. The means of public transport are poor. There are some cars that go to the villages but only once or twice per week. We have to rent motorcycles to do more home visits."*

### Recommendations for improvement of index testing

**Fig 4:**
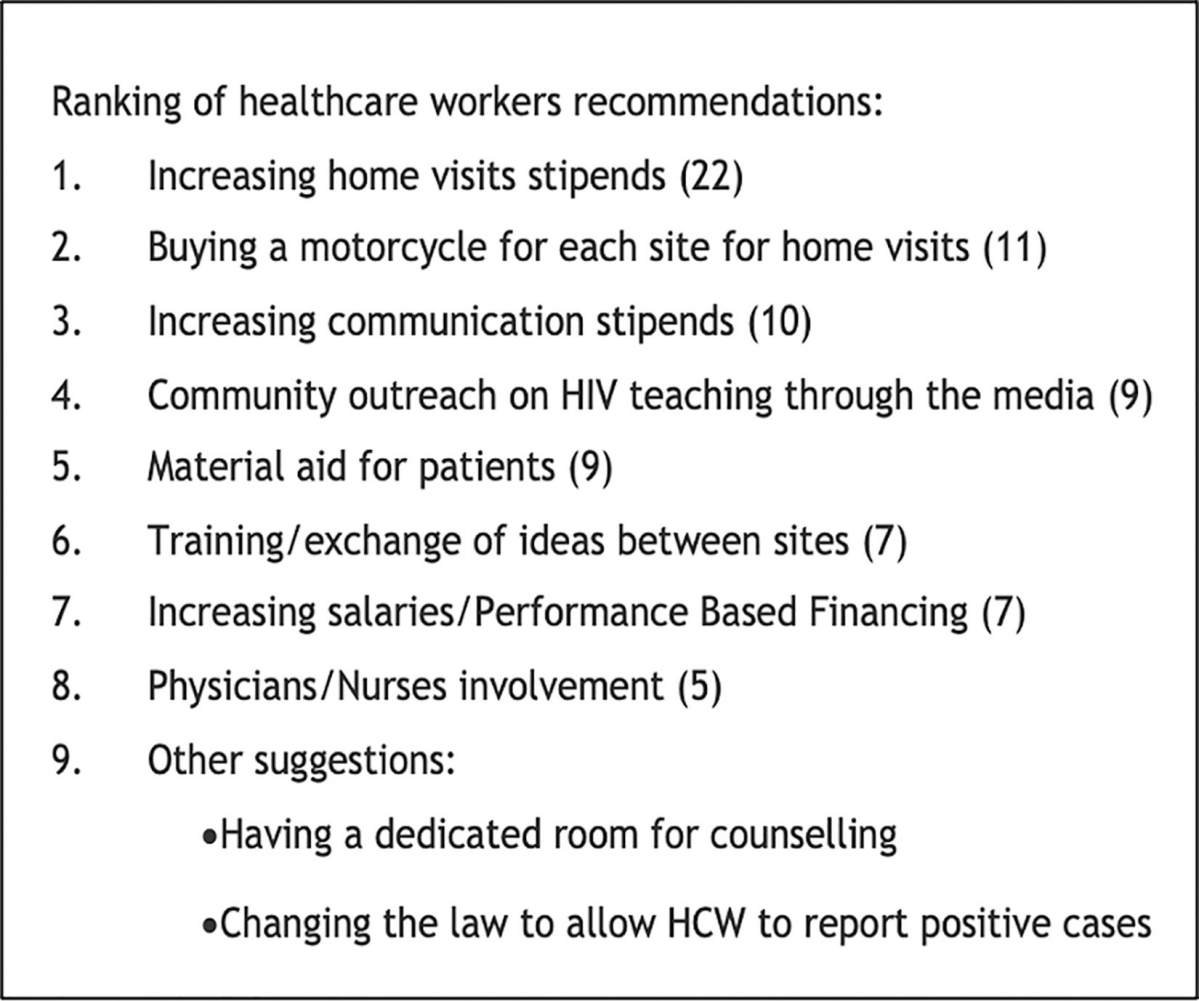
Ranking of community counselors’ recommendations regarding index testing.

Fig 4 lists the most common suggestions for improvement of the program gathered during the interviews. Given that the index testing process required repeated attempts to obtain names and execute contact tracing/ testing, the most prevalent themes were about improving the ability to do home visits and calls to patients. HIV education in the community, healthcare workers’ training, and building appropriate infrastructures were also among the recommendations offered by interview participants.

## Discussion

We found that passive and providers referrals are the preferred strategies, that there is no difference in practices of immediate versus delayed notification, and most prevalent challenges of index testing implementation reported by all providers relate to fear of stigma, fear of having marital problems, lack of training, and lack of infrastructures.

The finding of HCWs preferring passive or provider referral strategies is congruent with multiple other index testing studies [9,24–27]. A qualitative study done in Malawi reported passive notification to be the preferred method for HCW and index cases [24], whereas a similar study done in Kenya reported providers referral to be the preferred method for HCW [26]. In our study, the main reason reported for not offering the referral by contract was that this specific strategy was perceived as forcing the index case into the process. The responses of our participants implied that the referral by contract is not culturally appropriate in their communities. When it comes to the dual referral strategy, the reason for not using it was the lack of resources for doing calls or home visits due to distance, this was later brought up with their recommendations on how to improve index testing.

The preference of one strategy over another in each region raises the question of whether the lack of diversity in the strategies used may be contributing to lower case finding in Côte d’Ivoire. Results from a retrospective cohort study in Côte d’Ivoire showed that passive referral is most effective for children only, whereas providers referral is more effective for tracing and testing sexual partners [19]. If the results of the latter study are applicable to the regions that we investigated, then the sites where passive referral is preferred will have low yield of sexual partner tracing, which is the population that has a higher rate of positivity than children. In contrast, other studies have suggested that these referral strategies can be effective in eliciting and testing adult partners as stand-alone strategies [20,27]. The evidence suggests that the preference of one strategy over another can be successful. Based on this, this finding does not explain the low yield of index testing in Côte d’Ivoire.

In some cases, community counselors reported that the reason for not offering a variety of strategies to the index client was because they did not feel comfortable with the strategies due to a lack of in-depth training. The formal training was only implemented when the program started. As more community counselors were hired over the years, there was less in-depth training for each subsequently hired counselor. This observation highlights the need to implement formal training combined with subsequent review sessions on index testing strategies so that all community counselors are equally comfortable offering and explaining multiple strategies to their index clients regardless of their hire date.

Immediate versus delayed notification is also an important aspect of index testing. The responses from our interview participants did not convey a difference between the practice of one or another. A Kenyan study that compared immediate and delayed notification showed that immediate notification was more efficient than delayed notification in promoting HIV testing [28]. Another randomized controlled trial in Kenya found that case finding was higher in the intervention group with immediate notification than the control group with delayed notification, with an incidence rate ratio of 5 [29]. Not prioritizing immediate notification is may therefore be an important aspect that is influencing the results of index testing in Côte d’Ivoire.

The fact that non-clinical providers are the main implementors of index testing in Côte d’Ivoire is not surprising as task-shifting HIV services to lay providers has been a widespread strategy to mitigate the problem of HCWs shortage. In our study, the non-clinical providers expressed a dissatisfaction towards them being primarily responsible for carrying out all the responsibility of index testing as they did not feel fully equipped and trained to do so. Additionally, in terms of privacy, physicians and nurses usually have a private room for consultations whereas community counselors share rooms which ends up being a barrier to asking index testing related questions. There is evidence on the effectiveness HIV care task shifting as it relates to anti-retroviral initiation, medication adherence, or prevention of mother to child transmission, but to our knowledge, the subject of index testing task shifting has not been evaluated and would be worth looking into based on our findings.

**Fig 5:**
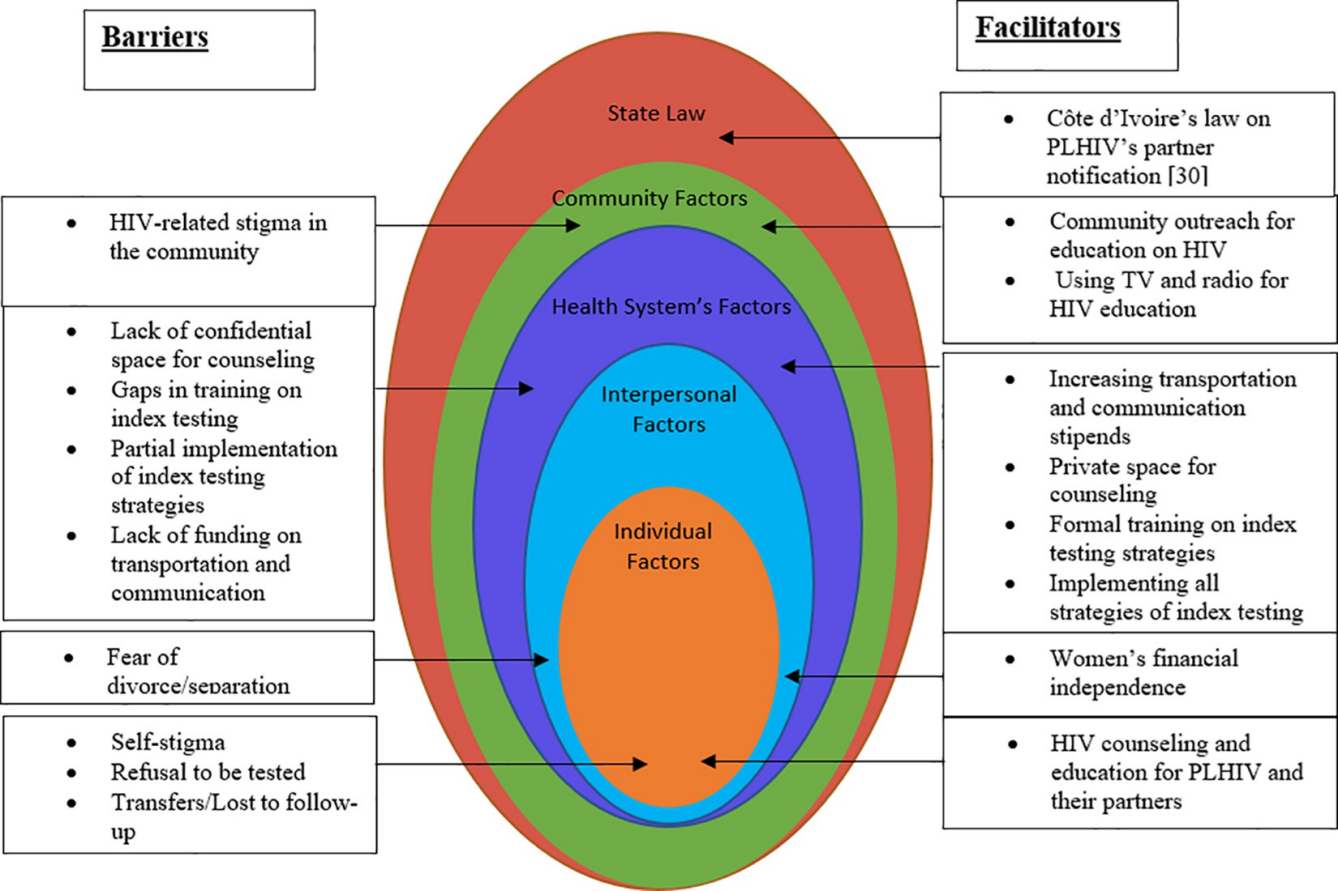
Barriers and facilitators of index testing. Summary of index testing barriers and proposed solutions (facilitators) using a socio-ecological model.

The reported challenges and suggestions for improvement relate to multiple layers of the socio-ecological model as illustrated in Fig 5.

At the root of some of the challenges reported appeared to be the persistent stigma in communities. HIV-related stigma in communities remains a significant obstacle to achieving the UNAIDS first 95% goal, as it hinders testing and the disclosure of one’s HIV-positive status to their family and friends [30,31]. The fear of divorce/separation and community level stigma-as obstacles to PLHIV signing up for index testing- were recurrent themes in our participants responses across all four regions. These barriers could be partly mitigated with community outreach to scale up HIV awareness through the media, skits at community events, dissemination of information through religious and other trusted community leaders. Several studies have shown that community outreach interventions are effective in reducing HIV-related stigma among populations. In a pre-and post-intervention survey in Thailand, researchers demonstrated that community participation in HIV education and sharing of opinions resulted in improvement in HIV knowledge and a reduction in stigma against PLHIV. There was a significant increase in both the HIV/ AIDS knowledge score (from a mean of 9.13 to 13.13) and the HIV/AIDS stigma score (from a mean of 98.75 to 133.84) [32]. School-based comprehensive HIV education has also reduced bias against PLHIV in studies conducted in Nigeria and Tanzania [33,34].

Some of the limitations of the study were that most healthcare workers surveyed (community counselors, supervisors) were staff of one NGO, HAI. Therefore, the results may not be generalized to HCW practices across the country. The lack of privacy noted above might have affected some of the answers as we had to conduct some interviews with other people in the room. Future studies should widen the sample of participants from centers funded by other NGOs.

## Conclusion

This study highlighted the complexity and challenges of index testing implementation in Côte d’Ivoire. It demonstrated its challenges at individual, interpersonal, and systems levels. Understanding the challenges of index testing implementation at each level of the socio-ecological model is important to implement effective, targeted efforts in order to improve the program, identify, and provide care to PLHIV who are unaware of their status. Addressing these challenges is not easy. However, interventions at any level of the socio-ecological model are likely to have a positive impact and would be worth the effort given the importance and documented effectiveness of index testing.

## Supporting information

S1 Fig When do you register names of index clients family and partners? (TIF)

S1 File Interview templates. (DOCX)
