# Supplementary figures and images for: Implementation process and challenges of index testing in Côte d’Ivoire from healthcare workers’ perspectives
Source: PLoS One. 2023 Feb 8;18(2):e0280623. doi: 10.1371/journal.pone.0280623 (PMC9907845; doi:10.1371/journal.pone.0280623)

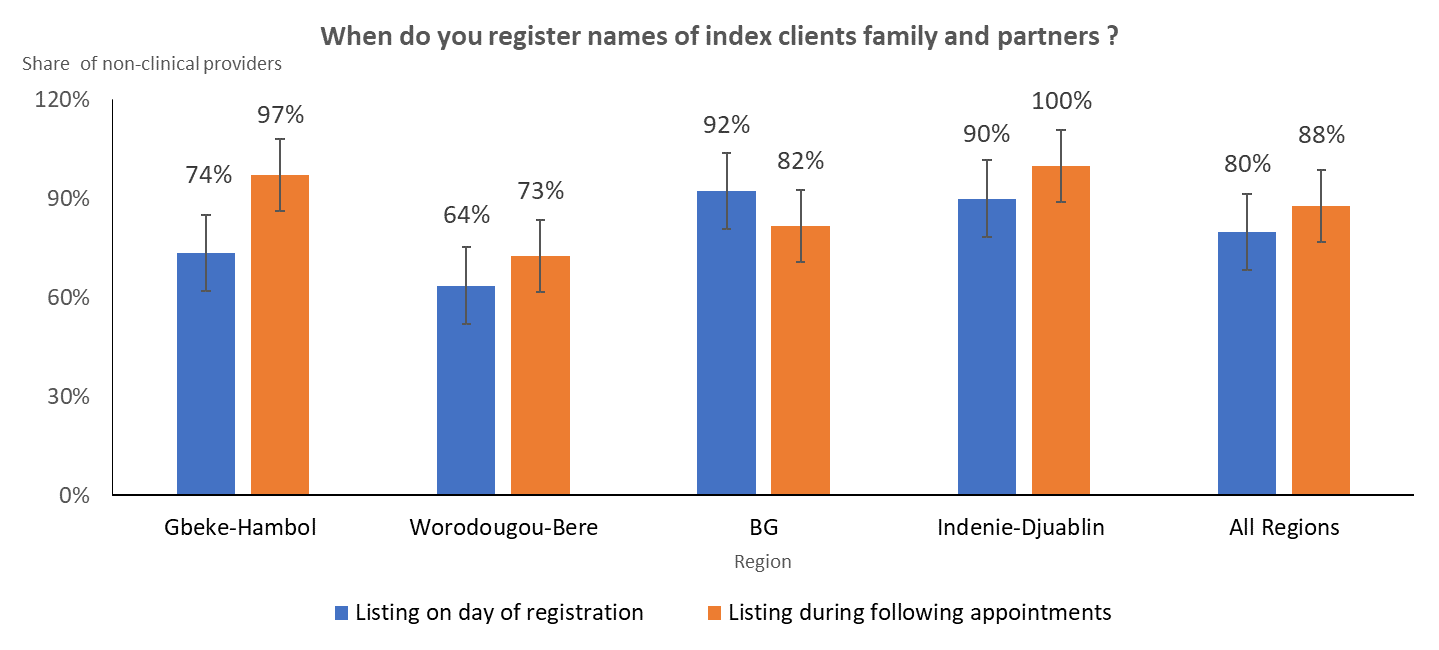

Supplement: S1 Fig — (TIF) [file pone.0280623.s001.tif]
