# Supplementary material for: Implementation process and challenges of index testing in Côte d’Ivoire from healthcare workers’ perspectives
Source: PLoS One. 2023 Feb 8;18(2):e0280623. doi: 10.1371/journal.pone.0280623 (PMC9907845; doi:10.1371/journal.pone.0280623)
Supplement: S1 File — (DOCX) [file pone.0280623.s002.docx]

**Supporting Information: Interview templates (Translated from French templates)**

**Participants: Community counselors, site supervisors**

1. How do you obtain the names and contacts of index clients' family members and sexual partners ?
2. Are there procedural guidelines on how to obtain and register those names?
3. When do you register names of index clients' families and partners?
4. Do you ask during subsequent visits if the index client has:
   1. A new spouse?
   2. A new sexual partner?
5. What are the strategies used for contact tracing and testing at your site?
6. Which strategy do you most often use?
   1. Passive referral?
   2. Provider's referral?
   3. Referral by contract?
   4. Dual referral?
7. If the index client chooses passive referral, how do you counsel them on bringing their family members and partners to be tested?
8. If the index client chooses the provider's referral, how do you reach out to their partners?
9. If the index client chooses the referral by contract, how much time do you provide for them to get their partners tested?
10. If the index testing chooses dual referral, how do you proceed to work with them on contact tracing and testing?
11. Do you do home visits?
12. What challenges have you encountered with obtaining the names of family and sexual partners?
13. What challenges have you encountered with testing family and sexual partners
14. Do you think your work setting respects confidentiality ?
15. Could you show me the documents you use for index testing ?
16. What suggestions do you have to improve the index testing program?

**Participants: Nurses/Physicians/Midwives**

1. Could you explain what steps are taken after a patient is diagnosed with HIV?
2. How are you involved in index testing?
3. Who tests patients when they present to the health center for HIV testing?
4. Who announces the results to the patient?
5. Who prescribes ART?
6. When do you ask a newly diagnosed patient to come back for a follow-up?
7. What do you think are the challenges of index testing at your site?
8. What are your suggestions to improve the index testing program?
